# Supplementary material for: Upregulation of the Renin–Angiotensin System Is Associated with Patient Survival and the Tumour Microenvironment in Glioblastoma
Source: Cells. 2024 Apr 5;13(7):634. doi: 10.3390/cells13070634 (PMC11012120; doi:10.3390/cells13070634)
Supplement: Supplementary file 1 [file cells-13-00634-s001.zip › Supplementary Figures.pdf]

A

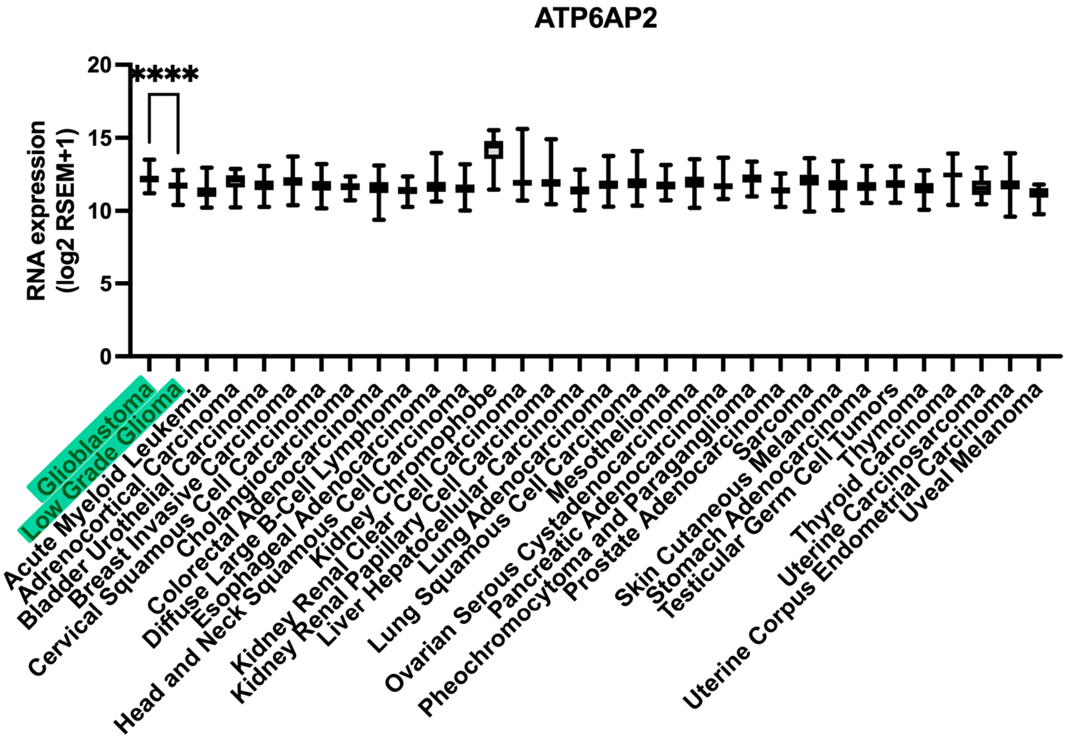
ATP6AP2
RNA expression
(log2 RSEM+1)
20
15
10
5
0
****
Glioblastoma
Low Grade Glioma
Acute Myeloid Leukemia
Adrenocortical Carcinoma
Bladder Urothelial Carcinoma
Breast Invasive Carcinoma
Cervical Squamous Cell Carcinoma
Cholangiocarcinoma
Colorectal Adenocarcinoma
Diffuse Large B-Cell Lymphoma
Esophageal Adenocarcinoma
Head and Neck Squamous Cell Carcinoma
Kidney Chromophobe
Kidney Renal Clear Cell Carcinoma
Kidney Renal Papillary Cell Carcinoma
Liver Hepatocellular Carcinoma
Lung Adenocarcinoma
Lung Squamous Cell Carcinoma
Mesothelioma
Ovarian Serous Cystadenocarcinoma
Pancreatic Adenocarcinoma
Pheochromocytoma and Paraganglioma
Prostate Adenocarcinoma
Sarcoma
Skin Cutaneous Melanoma
Stomach Adenocarcinoma
Testicular Germ Cell Tumors
Thymoma
Thyroid Carcinoma
Uterine Carcinosarcoma
Uterine Corpus Endometrial Carcinoma
Uveal Melanoma

B

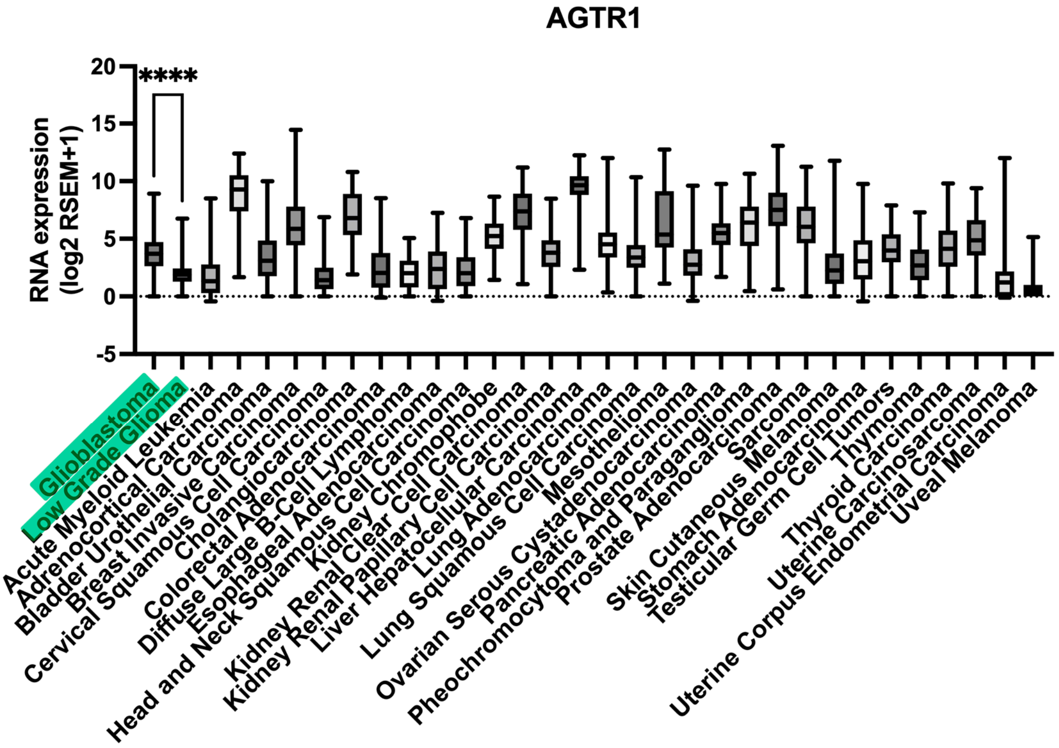
AGTR1
RNA expression
(log2 RSEM+1)
20
15
10
5
0
-5
****
Glioblastoma
Low Grade Glioma
Acute Myeloid Leukemia
Adrenocortical Carcinoma
Bladder Urothelial Carcinoma
Breast Invasive Carcinoma
Cervical Squamous Cell Carcinoma
Cholangiocarcinoma
Colorectal Adenocarcinoma
Diffuse Large B-Cell Lymphoma
Esophageal Adenocarcinoma
Head and Neck Squamous Cell Carcinoma
Kidney Chromophobe
Kidney Renal Clear Cell Carcinoma
Kidney Renal Papillary Cell Carcinoma
Liver Hepatocellular Carcinoma
Lung Adenocarcinoma
Lung Squamous Cell Carcinoma
Mesothelioma
Ovarian Serous Cystadenocarcinoma
Pancreatic Adenocarcinoma
Pheochromocytoma and Paraganglioma
Prostate Adenocarcinoma
Sarcoma
Skin Cutaneous Melanoma
Stomach Adenocarcinoma
Testicular Germ Cell Tumors
Thymoma
Thyroid Carcinoma
Uterine Carcinosarcoma
Uterine Corpus Endometrial Carcinoma
Uveal Melanoma

Figure S1 continued…

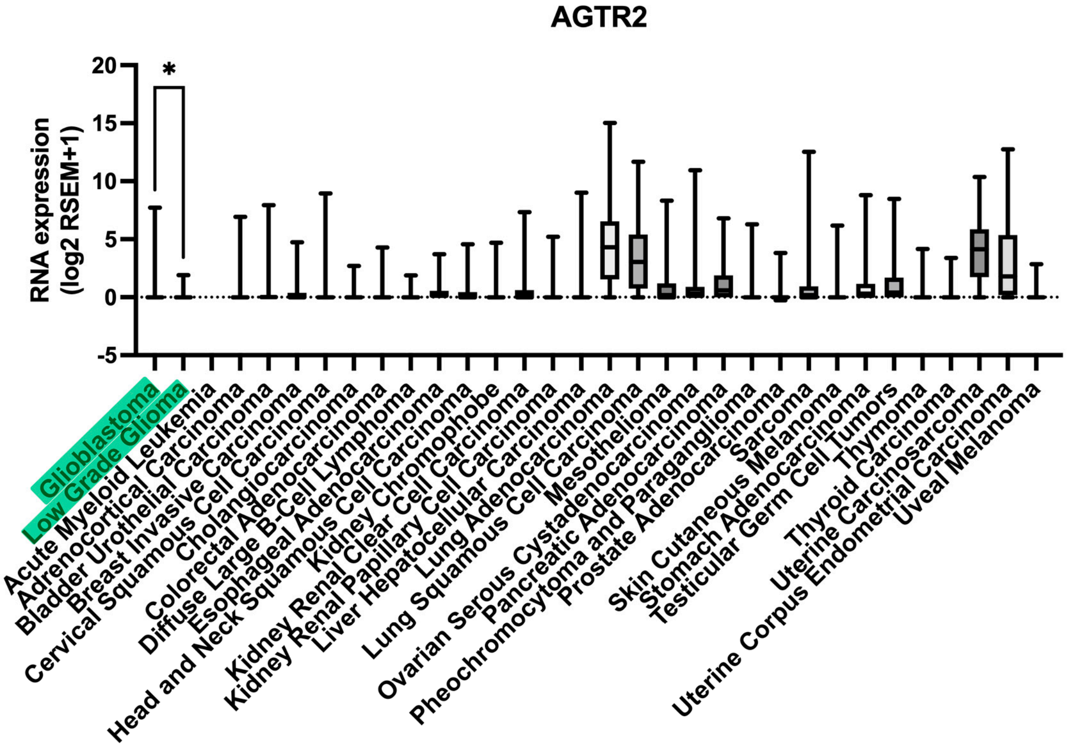

D

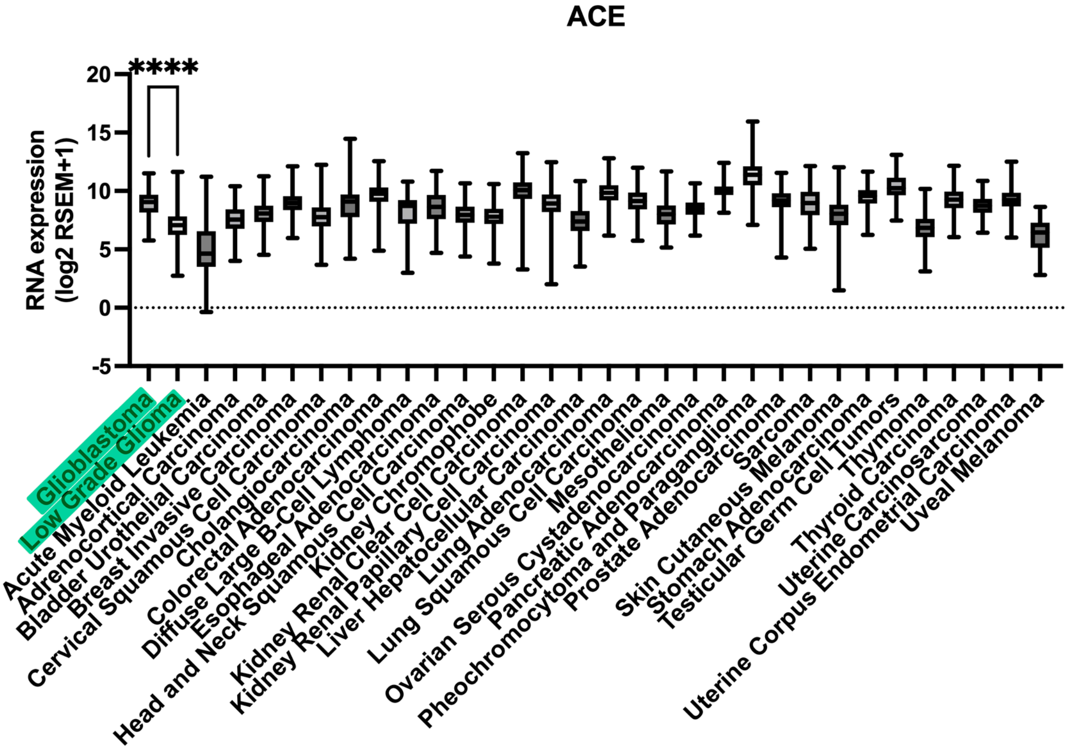

Figure S1 continued…

E

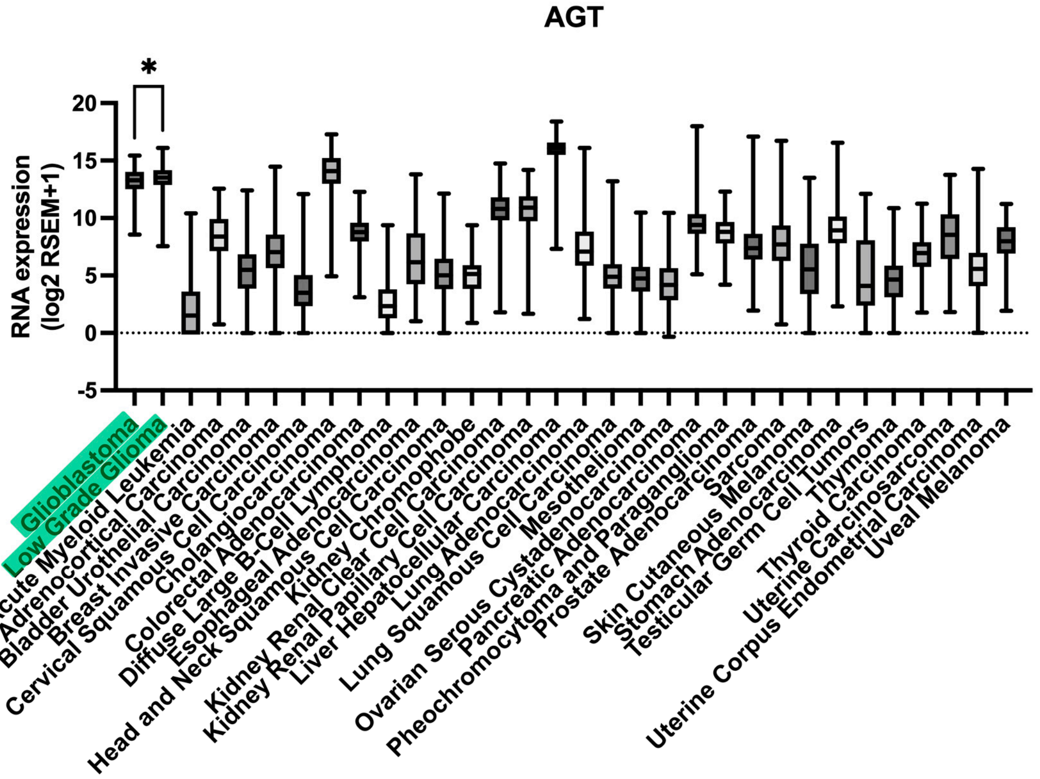

F

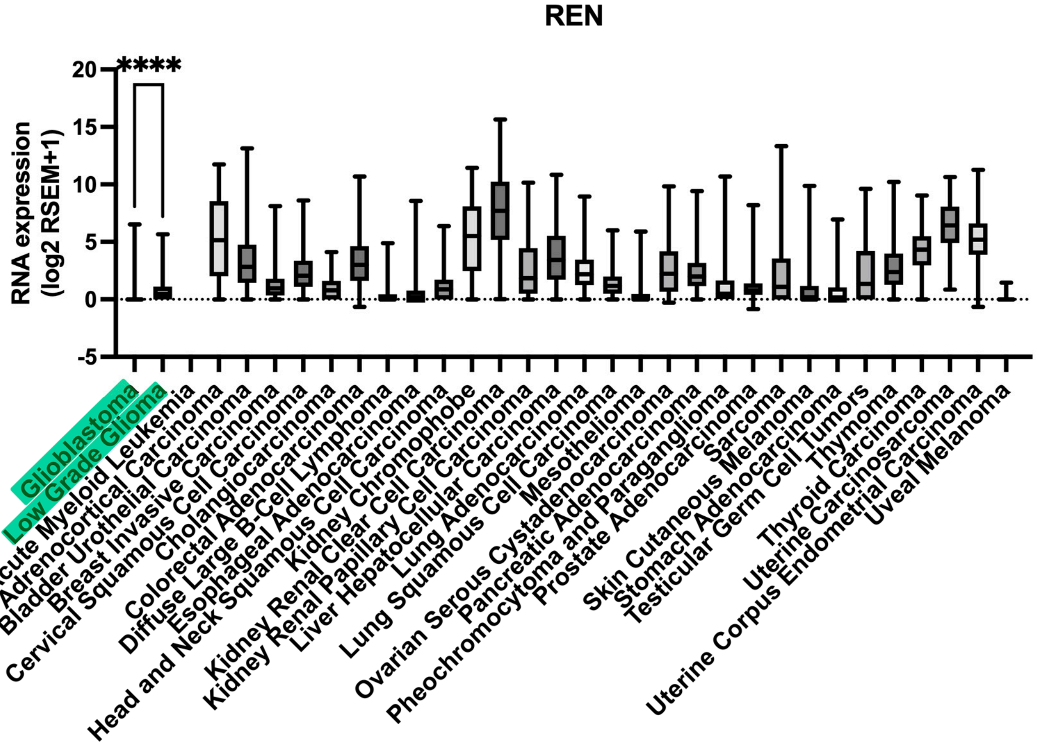

Figure S1. RAS gene expression across TCGA PanCancer tumours. RNA expression (log2 RSEM +1) of *ATP6AP2* (**A**) *AGTR1* (**B**), *AGTR2* (**C**), *ACE* (**D**), *AGT* (**E**) and *REN* (**F**) genes are shown across TCGA PanCancer patient samples. Data is represented as box plots. A Student's t-test was used to compare the levels of expression between the glioblastoma and LGG cohort (* $p < 0.05$, ** $p < 0.01$, *** $p < 0.001$, **** $p < 0.0001$), as also seen in Figure 1 of the main text. Glioblastoma and LGG are highlighted in green.

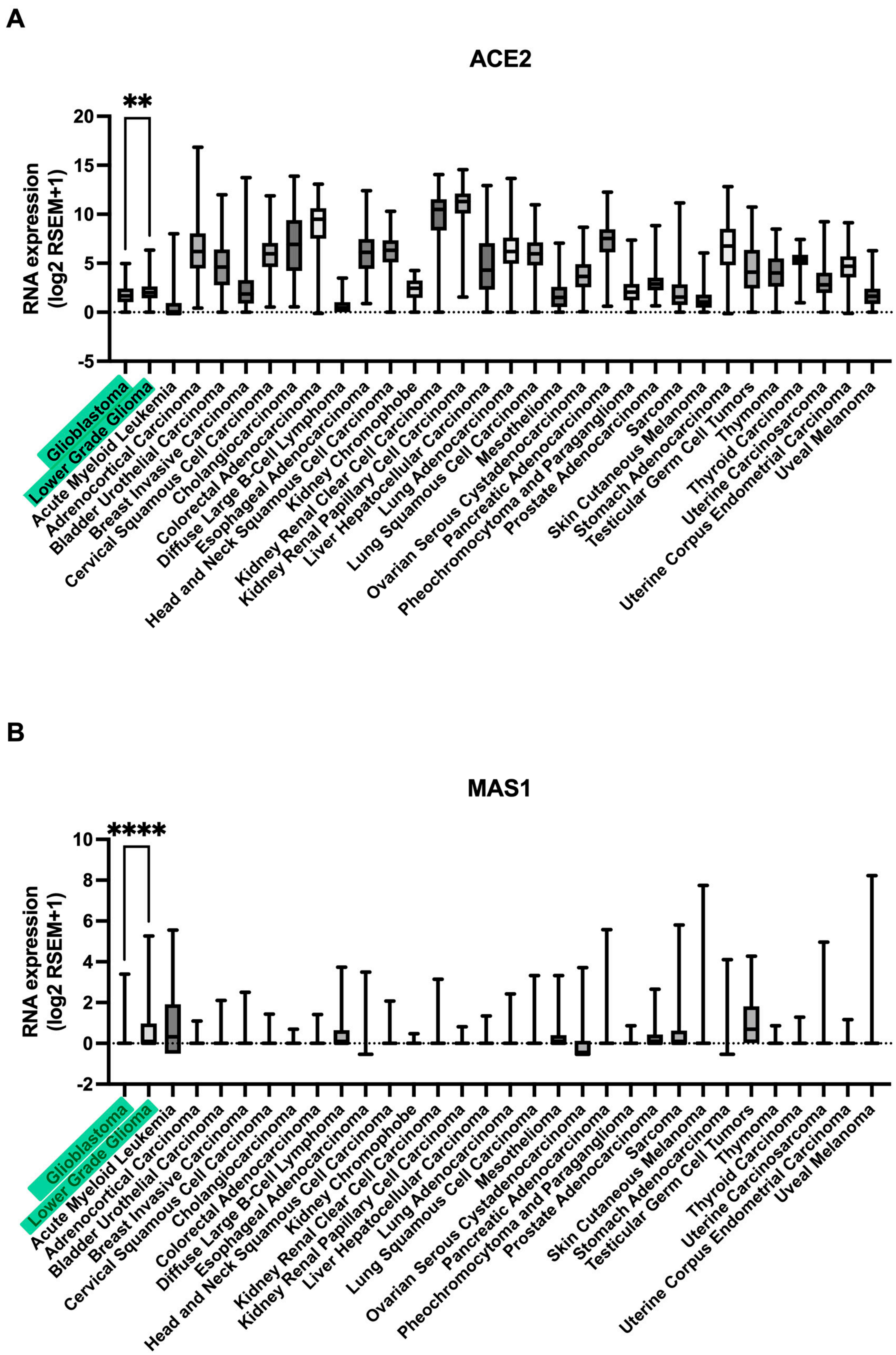

Figure S2. ACE2 and MAS1 gene expression across TCGA PanCancer tumours. RNA expression (log2 RSEM +1) of *ACE2* (**A**) and *MAS1* (**B**) are shown across TCGA PanCancer patient samples. Data is represented as box plots. A Student's t-test was used to compare the

levels of expression between the glioblastoma and LGG cohort (* $p < 0.05$, ** $p < 0.01$, *** $p < 0.001$, **** $p < 0.0001$). Glioblastoma and LGG are highlighted in green.

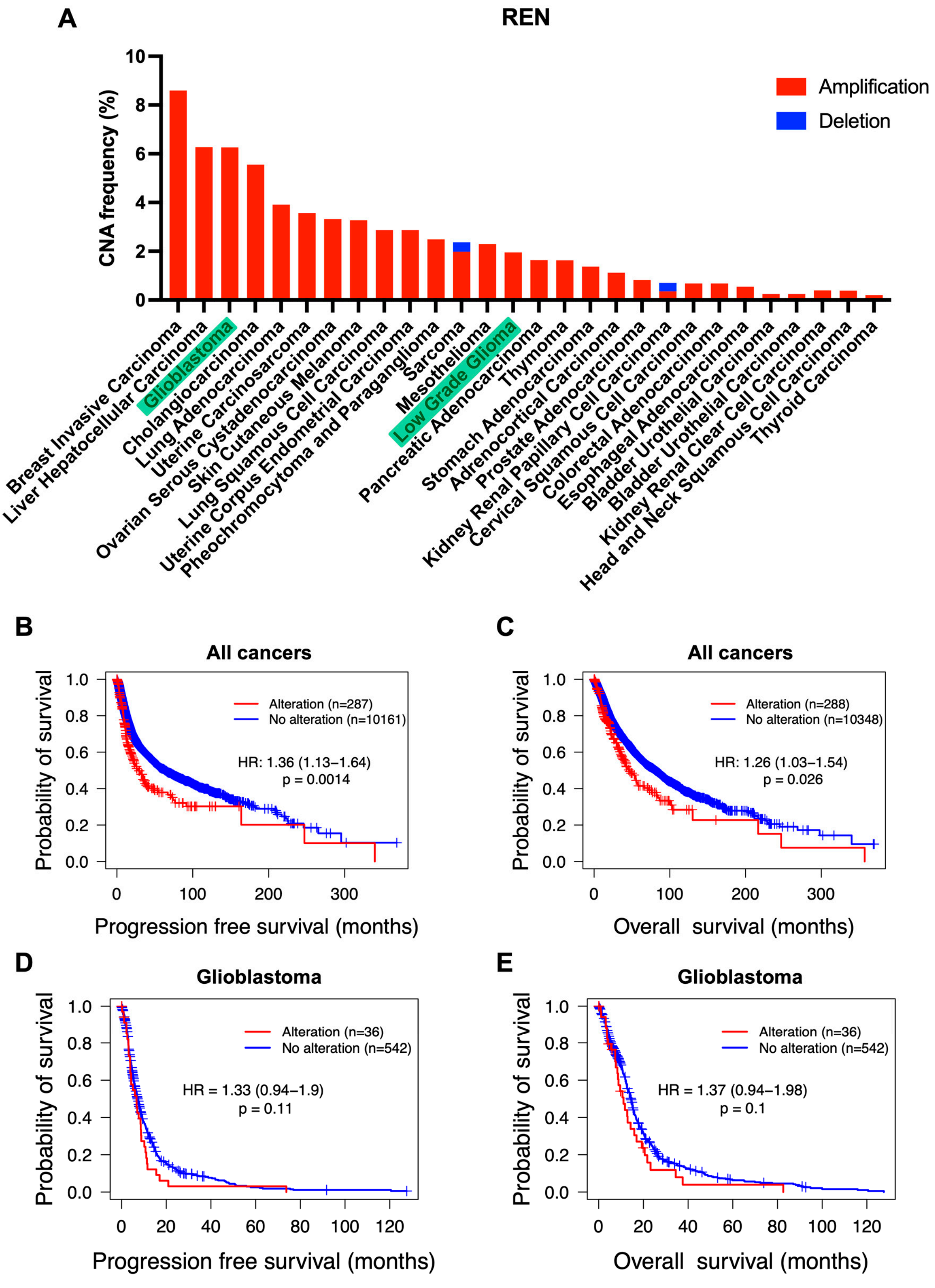

Figure S3. Copy number alterations (CNA) of REN across PanCancer tumours and survival outcomes. A) The frequency of CNA (amplification or deletion) are depicted across all cancer types from the TCGA PanCancer study, where glioblastoma (highlighted green) ranks the 3rd highest with 6% of samples displaying an alteration that contains amplifications of this gene. **B-C**) Kaplan-Meier plots are depicted for PFS and OS of all PanCancer cases containing CNA alterations or not, with hazard ratios and *p*-values shown after multivariate cox regression. Age, sex, and cancer type were significant clinical factors that were used in

multivariate cox analysis, with *REN* alterations significantly associated with poorer PFS and OS. **D-E**) Kaplan-Meier plots are displayed for PFS and OS of glioblastoma cases containing CNA alterations of *REN* vs no alteration. Hazard ratios and *p*-values are shown after univariate cox regression, where no significant difference was found for PFS or OS.

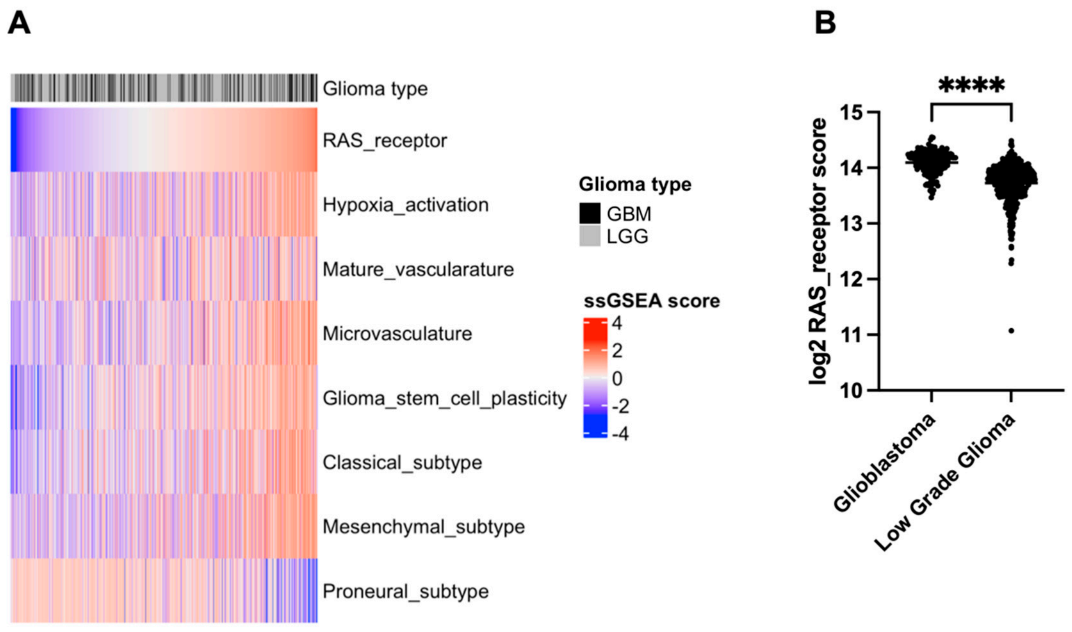

Figure S4. RAS receptor expression and its relationship to tumour microenvironment pathways in TCGA glioma cases. A) Log-transformed ssGSEA z-scores are represented for the RAS receptor pathway and TME-related pathways within glioma patient samples (glioblastoma and LGG). **B**) Comparison of RAS receptor ssGSEA score between glioblastoma and LGG cases within the TCGA. Statistical analysis was performed using an unpaired t-test (****$p < 0.0001$), with $p < 0.05$ considered significant.
